# The Need for Standardized Assessment of Muscle Quality in Skeletal Muscle Function Deficit and Other Aging-Related Muscle Dysfunctions: A Symposium Report

**DOI:** 10.3389/fphys.2017.00087

**Published:** 2017-02-15

**Authors:** Rosaly Correa-de-Araujo, Michael O. Harris-Love, Iva Miljkovic, Maren S. Fragala, Brian W. Anthony, Todd M. Manini

**Affiliations:** ^1^Division of Geriatrics and Clinical Gerontology, National Institute on Aging, National Institutes of Health, U.S. Department of Health and Human ServicesBethesda, MD, USA; ^2^Muscle Morphology, Mechanics and Performance Laboratory, Clinical Research Center – Human Performance Research Unit, Veterans Affairs Medical CenterWashington, DC, USA; ^3^Geriatrics and Extended Care Service/Research Service, Veterans Affairs Medical CenterWashington, DC, USA; ^4^Department of Exercise and Nutritional Sciences, Milken Institute School of Public Health, The George Washington UniversityWashington, DC, USA; ^5^Department of Epidemiology, Graduate School of Public Health, University of PittsburghPittsburgh, PA, USA; ^6^Quest DiagnosticsMadison, NJ, USA; ^7^Laboratory for Manufacturing and Productivity, Massachusetts Institute of TechnologyCambridge, MA, USA; ^8^Medical Electronic Device Realization Center, Massachusetts Institute of TechnologyCambridge, MA, USA; ^9^Department of Aging & Geriatric Research, Institute on Aging, University of Florida College of MedicineGainesville, FL, USA

**Keywords:** muscle quality, sarcopenia, muscle strength, muscle power, skeletal muscle function deficit, myosteatosis, imaging

## Abstract

A growing body of scientific literature suggests that not only changes in skeletal muscle mass, but also other factors underpinning muscle quality, play a role in the decline in skeletal muscle function and impaired mobility associated with aging. A symposium on muscle quality and the need for standardized assessment was held on April 28, 2016 at the International Conference on Frailty and Sarcopenia Research in Philadelphia, Pennsylvania. The purpose of this symposium was to provide a venue for basic science and clinical researchers and expert clinicians to discuss muscle quality in the context of skeletal muscle function deficit and other aging-related muscle dysfunctions. The present article provides an expanded introduction concerning the emerging definitions of muscle quality and a potential framework for scientific inquiry within the field. Changes in muscle tissue composition, based on excessive levels of inter- and intra-muscular adipose tissue and intramyocellular lipids, have been found to adversely impact metabolism and peak force generation. However, methods to easily and rapidly assess muscle tissue composition in multiple clinical settings and with minimal patient burden are needed. Diagnostic ultrasound and other assessment methods continue to be developed for characterizing muscle pathology, and enhanced sonography using sensors to provide user feedback and improve reliability is currently the subject of ongoing investigation and development. In addition, measures of relative muscle force such as specific force or grip strength adjusted for body size have been proposed as methods to assess changes in muscle quality. Furthermore, performance-based assessments of muscle power via timed tests of function and body size estimates, are associated with lower extremity muscle strength may be responsive to age-related changes in muscle quality. Future aims include reaching consensus on the definition and standardized assessments of muscle quality, and providing recommendations to address critical clinical and technology research gaps within the field.

## Introduction

A symposium was convened at the 2016 International Conference on Frailty and Sarcopenia Research (April 28, 2016 in, Philadelphia, PA) to discuss the impact of muscle quality on age-related muscle dysfunction and to explore approaches to standardized assessment. Adverse changes in muscle tissue composition have long been acknowledged as a contributor to the diminished muscle performance observed in older adults (Goodpaster et al., 2000; Manini et al., 2007). However, the routine assessment of this muscle characteristic has been limited due to a combination of factors. These factors may include relatively high costs, access limitations in some practice environments, and the testing burden associated with invasive techniques. Importantly, the wider adoption of routine tissue composition analysis is constrained by the need for additional evidence to demonstrate its value in the diagnosis of age-related muscle dysfunction, and utility in supporting diagnostic and therapeutic decisions. Growing evidence does suggest that age-related increases in intramuscular adipose tissue may compromise muscle performance and metabolic status (Miljkovic and Zmuda, 2010). Efforts to integrate these clinically relevant research insights into practice have driven the development of alternative assessment methods. Clinically viable modalities ranging from multi-frequency electrical impedance analysis to quantitative diagnostic sonography have been recently developed to characterize skeletal muscle mass and quality in older adults and those with muscle disease (Harris-Love et al., 2014). There has also been increasing interest in non-invasive, high precision, imaging modalities. For example, magnetic resonance imaging (MRI) has been used for the diagnosis and assessment of disease progression for a number of neuromuscular diseases such as Duchenne muscular dystrophy (Finanger et al., 2012). In addition, investigators have increasingly integrated the concepts of peak force production and body size to assess muscle performance and provide an estimate of muscle quality (Cawthon et al., 2009; Fragala et al., 2015b). Consequently, the selected symposium topics included a brief review of potential mechanisms that adversely affect muscle quality, and developing imaging methods and performance-based tests used to assess muscle quality within a variety of settings. The symposium presentations, and subsequent panel discussion, highlighted the need for continued dialogue concerning the definition and taxonomy used to describe muscle quality. Therefore, the primary aims of this symposium report are to introduce a potential framework for the study of muscle quality, convey the metabolic and functional consequences of poor muscle tissue composition, review a functional approach to assessment that incorporates relative strength, and examine emerging sonographic methods used to characterize muscle tissue.

## Developing a framework for assessing muscle quality

The age-related decline of skeletal muscle mass and its function is associated with physical limitations, fall risk, disability, and mortality in adults over the age of 60 (Janssen et al., 2002; Landi et al., 2013; Charlier et al., 2015; Clynes et al., 2015; Brown et al., 2016). These mobility impairments are often a precursor of functional decline, disability, and loss of independence. Consequently, sarcopenia continues to pose challenges to older adults, their caregivers, and the U.S. healthcare system (Janssen et al., 2004b). The decrease in lean body mass (LBM) associated with sarcopenia often results in diminished muscle strength or power (Morley, 2012). Peak muscle force as a function of muscle size is a well-established precept in physiology (Bamman et al., 2000). The observations by James Keill in the early 1700s established that muscle strength corresponds to the number of associated muscle fibers (Keill, 1708), and Ernst Weber (Narici et al., 2016) investigated the relationship between maximum isometric force and muscle cross-sectional area (CSA) in 1846. Importantly, adverse changes in muscle quality, based on tissue composition or peak force production, may precede a loss of muscle mass. Evidence now shows that not only changes in skeletal muscle mass, but other factors underpinning muscle quality may play a role in the decline in skeletal muscle function and impaired mobility associated with aging (Visser et al., 2005; Goodpaster et al., 2006; Ferrucci et al., 2011). While muscle size appears to contribute to losses in strength and physical functioning, both the response to strength training and the consequences of chronic disuse involve physiologic adaptations that are intrinsically separate from skeletal muscle size (Chilibeck et al., 1998; Clark et al., 2008). Moreover, skeletal muscle tissue has varied physiologic roles that impart wide ranging effects on multiple body systems. Therefore, careful consideration should be given to the operational definition of “muscle quality” since its interpretation is affected by the specific physiologic function of interest and the methodology used to characterize tissue or measure performance.

Muscle quality has been described in myriad ways by clinicians and investigators. *Hazzard's Geriatric Medicine and Gerontology* aptly conveys the broad concept of muscle quality with a description that includes glucose metabolism, oxidative damage, protein metabolism, intramuscular adipose tissue, capillary density, structural composition, contractility and fatigability (Hazzard and Halter, 2009). Furthermore, those that conduct sarcopenia-related studies have frequently used relative force production (expressed as a ratio of peak force and a measure of body size, regional LBM, or cross-sectional area) as a preferred approach to characterizing muscle quality (Cawthon et al., 2009; Scott et al., 2010; Kennis et al., 2014). The term, muscle quality—despite its ambiguity—has served the useful purpose of allowing investigators to explore facets of skeletal muscle function deficit beyond the construct of the age-related decline of LBM. Uniformity and clarity, however, are needed regarding the definition of muscle quality and the measurement methodology. The general definition of *quality* incorporates an object's “essential character” and its “distinguishing attributes,” and may also include a comparative “degree of excellence” (Merriam-Webster, 2004). Thus, the inherent meaning of muscle quality is inextricably linked to the primary functions of skeletal muscle, and this meaning may be further expanded to consider normal physiology vs. pathophysiology. The term, skeletal muscle function deficit (SMFD), was coined by Correa-de-Araujo and Hadley (2014) to encompass the evolving concepts of sarcopenia and other aging-related muscle dysfunction that contribute to clinically meaningful mobility impairments (Correa-de-Araujo and Hadley, 2014). In considering other conditions that are clinically manifested as impaired physiologic functions and have multiple contributory factors (e.g., congestive heart failure and chronic obstructive pulmonary disease), this type of diagnostic evolution may accommodate both therapeutic progress at a stage when mechanistic information has been limited, and further progress as mechanistic understanding continues to increase. Therefore, in building a framework for muscle quality, it is critical to note the complexity of skeletal muscle tissue and its physiologic roles that include not only movement via force production, but also metabolism through its maintenance of glucose/insulin homeostasis and amino acid storage (Miljkovic and Zmuda, 2010), thermoregulation, and autocrine, paracrine, and endocrine signaling via myokine production (Pedersen, 2011; Ahima and Park, 2015; Colaianni and Grano, 2015). Considering this expansive view of muscle quality is essential to improving our understanding of SMFD. Muscle quality would then be ultimately determined by the degree that muscle tissue fulfills its roles. This includes function while at rest and in response to increased demands, within the limits of normal physiological capacity and reserve.

A proposed conceptual model for muscle quality may be built upon on the primary physiologic functions of muscle tissue and categorized in domains: *force production, metabolism, thermoregulation, signaling/myokine production*. Although muscle force production is perhaps the most prominent characteristic of muscle tissue, this observation does not mitigate the relative importance of skeletal muscle for glucose/insulin homeostasis and other biological functions. While the term “muscle quality” resists easy characterization, it shares many of the well-documented limitations of terminology used to describe the broad construct of muscle performance (Kluger et al., 2013). Many endpoint variables ranging from specific force to the proportional measures of intermuscular fat have been used to quantify muscle quality. Selecting ideal endpoint measures to better understand the muscle quality domains are dependent on the following factors: (1) established measurement analytics (e.g., validity, reliability, and responsiveness), (2) a meaningful association with a major domain of muscle quality, and (3) clear utility for research and/or clinical purposes. This conceptual orientation would be appropriate for both mechanistic and clinical approaches to characterize muscle quality and pursue specific lines of hypothesis-driven research. While no ideal singular endpoint measure is suitable to characterize muscle quality, consensus could be attained to recommend standardized approaches to endpoint measures within each of the major domains of muscle quality. These recommended endpoints should be categorized based on their suitability for either research settings or clinical environments. Subsequent research will determine the relative importance of selected endpoint measures of muscle quality. Investigators will utilize outcomes that are the most strongly associated with mechanisms that govern muscle performance or predict mobility status and mortality in older adults. This view of muscle quality further untethers investigators from a strict LBM-driven assessment approach to sarcopenia research, and may help to end the conflation between “measurement methods” and the targeted “domain” of muscle quality. This perspective is reflected in the ongoing research concerning the local and systemic effects of myosteatosis, and emerging methods to quantify changes in muscle tissue composition and function.

## Effects of myosteatosis on metabolic status

Muscle architecture, composition, metabolism, fat infiltration, fibrosis, and neural activation are among the multiple factors potentially influencing muscle quality (McGregor et al., 2014). Over the past decade, myosteatosis, the ectopic fat infiltration in skeletal muscle, has emerged as an important factor underpinning muscle quality and also as a possible predictor of muscle function and metabolic status (Miljkovic and Zmuda, 2010). Myosteatosis is a unique ectopic fat depot with broad clinical sequelae including poor metabolic and musculoskeletal health, accelerated aging and impaired longevity (Goodpaster et al., 2000, 2001, 2003; Visser et al., 2005; Miljkovic and Zmuda, 2010; Wijsman et al., 2012; Miljkovic et al., 2013b). Essentially, two modalities of fat depots are identified within skeletal muscle with varying functions (Vettor et al., 2009; Miljkovic and Zmuda, 2010): (1) Intermuscular fat defined as the visible extracellular adipose tissues located beneath the muscle and between and within muscle groups (Manini et al., 2007); (2) Fat infiltration within myocytes (known as intramuscular fat or intramyocellular lipids), which represents the microscopic lipid droplets utilized as energy within the muscle. Ectopic fat deposition in skeletal muscles progressively increases with aging, and seems to act synergistically with sarcopenia (Narici and Maffulli, 2010; Budui et al., 2015). Myosteatosis is also a finding common to muscular dystrophies and aging muscles.

### Local and systemic effects of myosteatosis

#### Myosteatosis and muscle and mobility function

**Figure 1:**
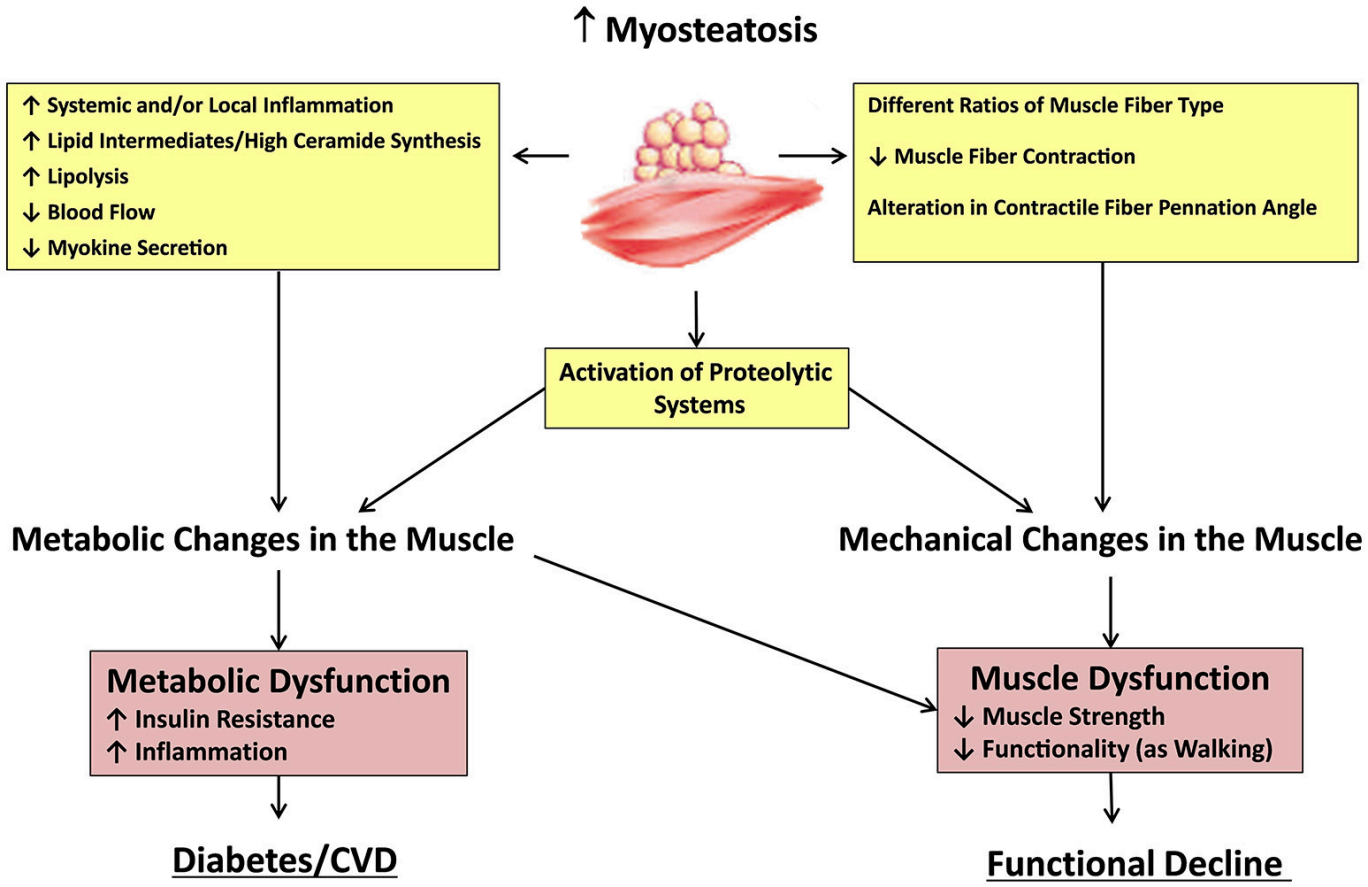
**Potential mechanisms underlying the effects of myosteatosis**. Increased myosteatosis may lead to metabolic and mechanical changes in the muscle through a variety of mechanisms. Changes in muscle cell metabolism can lead to increased insulin resistance and inflammation, aiding in the development of diabetes, and cardiovascular diseases. Alterations in muscle architecture can also lead to muscular dysfunction and functional decline. Both processes may be increased through activation of proteolytic systems, which may also result from increased myosteatosis.

Myosteatosis is not an inert fat depot that simply fills the space left by lean mass loss. Excessive levels of myosteatosis may negatively affect skeletal muscle tissue. For example, in young healthy individuals subjected to 30 days of leg disuse by suspension, myosteatosis increased 15–20% and exceeded the loss of lean calf and thigh mass (Manini et al., 2007). Myosteatosis may affect the muscle performance and physical mobility (Figure 1). High levels of myosteatosis are also associated with decreased activation of the quadriceps muscles in older adults (Yoshida et al., 2012a). Increased mobility loss, reflected by decreased 6-min walk distance (Marcus et al., 2012), decreased gait speed (Visser et al., 2002), decreased physical performance, difficulty with repeated chair stands (Visser et al., 2002), and slower stair descent and timed get-up and go tests (Marcus et al., 2012), is also the result of the effects of myosteatosis on muscle metabolism and function. Myosteatosis may also lead to the transition of muscle fibers from type II to type I (Mastrocola et al., 2015). This transition would result in muscles with impaired contractile capacity and decreased power (Reid and Fielding, 2012). Future studies are needed to elucidate the mechanisms (i.e., metabolic or mechanical changes) behind the increased myosteatosis and decreased muscle and mobility function. These studies should determine if minimizing myosteatosis is accompanied by improved muscle and mobility function (Goodpaster et al., 2001; Visser et al., 2005; Manini et al., 2007; Marcus et al., 2012; Tuttle et al., 2012; Yoshida et al., 2012a).

#### Myosteatosis and metabolism

There is a growing body of research on myosteatosis and its systemic effects related to metabolic disorders. It is known that independent of total body adiposity, measured either by body mass index (BMI) or dual energy X-ray absorptiometry (DXA) whole body fat, myosteatosis has been associated with insulin resistance and an increased risk of developing type-2 diabetes (Goodpaster et al., 2003; Miljkovic et al., 2013a, 2016), hypertension (Ding et al., 2004; Miljkovic et al., 2013a; Therkelsen et al., 2013), and dyslipidemia (Miljkovic et al., 2013b), usually detectable much earlier than the onset of metabolic disorders (Savage et al., 2007). The link between myosteatosis and metabolic disorders could be theoretically attributed to the relationship of myosteatosis and overall adiposity. With increasing adiposity, there is also an increase of the fat infiltration in the muscle (Gallagher et al., 2005). However, the association between myosteatosis and metabolic risk factors remains strong even after accounting for BMI, suggesting that these metabolic impairments are not simply due to obesity alone. The findings on the association between myosteatosis and metabolic disease independent of visceral fat have been inconclusive; it is unclear as of yet whether myosteatosis is associated with metabolic disorders when visceral and other ectopic fat depots are taken into account.

The metabolic consequences of myosteatosis depend on: (1) age, (2) race/ethnicity, (3) aerobic fitness levels, (4) sensitivity to insulin, (5) amount of physical activity, and (6) anatomical region. Myosteatosis has been found to increase with age, and the deposition of ectopic fat accelerates over time (Delmonico et al., 2009). In addition, compared with Caucasians, African ancestry individuals and South Asians may have more myosteatosis independent of differences in general adiposity (Miljkovic et al., 2009). Interestingly, marked increases in myosteatosis can have both adaptive and maladaptive effects on muscle function and metabolism. The “Athlete's paradox” is a phenomenon observed in endurance-trained athletes, who possess higher intramyocellular lipid content, coupled to high oxidative capacity and enhanced insulin sensitivity (Dubé et al., 2008). Athlete's paradox clearly demonstrates that intramyocellular fat does not always have deleterious effects and that there may be other “bad” lipids in muscle. Finally, due to a different function and morphology of skeletal muscles, increased fat infiltration in certain muscles may be found in normal physiologic conditions and may not have a harmful effect on metabolism. For example, under normal physiologic conditions, myosteatosis is lower in the tibialis muscle but higher in the soleus (Goodpaster et al., 2004; Lawrence et al., 2010). It is currently unknown whether myosteatosis acts merely as a marker of metabolic dysfunction or whether it may have an intermediary or modifying role in insulin resistance. More mechanistic and epidemiologic studies are needed to elucidate these questions (Gallagher et al., 2005; Yim et al., 2007; Miljkovic-Gacic et al., 2008; Boettcher et al., 2009; Ryan et al., 2011).

### Potential determinants of myosteatosis

Despite the growing clinical relevance of myosteatosis, the biological mechanisms underlying increases in myosteatosis with aging in humans remain considerably unknown. Current theories are largely based on animal models or controlled experimental studies in small samples of humans. Some propose that increased myosteatosis may result from increased fatty acid transport, uptake and storage, and reduced fatty acid oxidation (Kim et al., 2000; Kelley and Goodpaster, 2001). Other experiments suggest that macrophage infiltration inhibits adipocyte differentiation, and leads to adipocyte hypertrophy, altered cytokines secretion, and increased fat accumulation in muscle and other non-adipose tissues (Hegarty et al., 2002). It is also thought that an excess lipid availability and high-fat diet may reduce the mitochondrial respiratory chain components in muscle. Impaired skeletal muscle mitochondrial function, which directs fatty acids toward storage as opposed to oxidation, may also contribute to aging-related increases in skeletal muscle fat accumulation (Roden, 2005; Mathieu et al., 2010). Another potential mechanism for these alterations involves the possible age-related change in activation, proliferation, and differentiation of quiescent skeletal muscle precursor “stem” cells (i.e., satellite cells) into adipocytes in response to a range of stimuli that occurs with aging. Inadequate storage of excess fat in subcutaneous fat depots during excess lipid availability has also been identified as a potential cause of the increased myosteatosis. The subcutaneous fat depot may act as a metabolic “sink,” which can accommodate excess lipids and thus prevent the flow of lipid to vital organs. A better understanding and identification of the biological factors that regulate myosteatosis with aging, including the evidence of senescent cells and cultured cells developing into preadipocytes and fat cells (Tchkonia et al., 2010), is imperative for developing new strategies for promoting metabolic and musculoskeletal health. Such biomarkers could become targets for novel therapies to preserve a more functionally and metabolically “healthy” skeletal muscle with aging (Mathieu et al., 2010; Tchernof and Després, 2013; Smith, 2015).

### Potential mechanisms underlying the effects of myosteatosis

Though the specific mechanisms underlying the detrimental effects of myosteatosis on local and systemic muscle metabolism remain unknown, several hypotheses have been proposed that support this relationship (Albu et al., 2005; Gallagher et al., 2005; Durheim et al., 2008; Lee et al., 2009; Vettor et al., 2009; Kovalik et al., 2011; Trayhurn et al., 2011). It is theorized that the close proximity of fat to the muscle fiber may impair the local muscle environment through elevated levels of local or systemic pro-inflammatory cytokines (Vettor et al., 2009; Zoico et al., 2010) or throughout the impaired myokine secretion (Brandt and Pedersen, 2010). Increased myosteatosis may also impair nutritive blood flow to muscle and thus impair insulin action and insulin diffusion capacity (Lee et al., 2009). Additionally, recent findings indicate that an increased number of inter-muscular adipocytes have a direct local impact on skeletal myocyte metabolism, causing enhanced myotube mRNA expression of genes involved in oxidative metabolism (Lowell and Shulman, 2005; Kovalik et al., 2011). This study suggested that even a relatively small amount of intermuscular fat may be sufficient to drive myotubes into lipid oxidation and affect skeletal muscle metabolism. However, these effects depended on the metabolic state of the system. It is also hypothesized that the increasing rate of lipolysis within skeletal muscle results in an elevated concentration of glucose within the skeletal muscle (Yim et al., 2007; Turcotte and Fisher, 2008). From animal models, it has been shown that insulin resistance observed in old high-fat diet fed rats is independently related to myosteatosis, and that it can be induced by high ceramide synthesis (Tardif et al., 2014), stimulated by adipocyte-derived cytokines (including tumor necrosis factor-α; Summers and Nelson, 2005; Tardif et al., 2014) and consequent decrease in the ability to upregulate muscle insulin pathways.

In addition to the possible effects of myosteatosis on muscle and mobility function through changes in local muscle metabolism, myosteatosis may also be harmful to muscle and mobility function due to mechanical changes in muscle that occur in the presence of myosteatosis, leading to changes in muscle fiber orientation (Yoshida et al., 2012a). Along with the loss of elasticity in rotator cuff muscles, it has been hypothesized that excess myosteatosis leads to an alteration in contractile fiber pennation angle, hence resulting in an unfavorable mechanical angle and a concomitant reduction in force production (Meyer et al., 2004; Gerber et al., 2007). To our best knowledge, no studies have examined the effect of myosteatosis on elasticity or of pennation angle in locomotor muscles. Finally, pro-inflammatory cytokines (Vettor et al., 2009) secreted by adipose tissue in the skeletal muscle microenvironment may lead to proteolysis and muscle catabolism (Ebisui et al., 1995; Hegarty et al., 2002; Zoico et al., 2010). Figure 1 summarizes the potential mechanisms underlying the effects of myosteatosis.

### Imaging measures of myosteatosis

Image-based methods of assessing myosteatosis are applied primarily in research settings, and are not yet in routine clinical use. Based on these facts, there is a need to establish an objective, valid, reliable, and standardized measure of quality derived from the composition of the skeletal muscle. MRI of a cross section of the thigh of young and old males and females matched for weight and height demonstrate that fat infiltration is not an issue of being overweight, but a sign of important changes with aging (Petrella et al., 2007). MRI is an excellent non-invasive technique for measuring myosteatosis, providing high-quality images. However, the cost is high, and traditional MRI does not typically allow quantification of the fat content of the muscle. Recent MRI work is aiming to assess myosteatosis using a method for fat/water separation. Chemical shift-based MRI has demonstrated an advantage over single voxel spectroscopy in readily determining tissue heterogeneity within muscle fat fractions in both normal and dystrophic muscles (Triplett et al., 2014).

Other imaging techniques also facilitate the non-invasive evaluation of myosteatosis. Computed tomography (CT) has thus far probably been the most utilized as a research tool to investigate myosteatosis. It is basically assessed indirectly using muscle attenuation calculated CT imaging scan, leading to close correlation with direct measurements of muscle lipid content (Machann et al., 2003; Larson-Meyer et al., 2006). Muscle radiation attenuation is a radiological characteristic that can be measured (Goodpaster et al., 2000; Goodpaster, 2002). Any given skeletal muscle displays radiation attenuation between −190 and +150 Hounsfield units (HU), with a prominent peak near +50 HU. When muscle cross-sectional area and attenuation are reported, the most common practice is to use pre-established HU ranges to define intermuscular fat (usually −190 to −30 HU) and muscle tissue (usually −29 HU to 150 HU; Aubrey et al., 2014). Although radiation exposure is a disadvantage, compared to CT scans of the trunk, the effective dose to the extremities is typically very small. CT also cannot isolate individual muscle groups. New dual or multispectral CT imaging methods may offer improved tissue discrimination in future studies.

Standard MRI, CT, and DXA imaging are techniques frequently utilized in large clinical trials, but one of their major limitations is that they cannot directly measure the lipid content or detect the location of fat storage within myocytes (intramyocellular fat) or lipid droplets surrounding myocytes (extramyocellular fat, which are present in very small quantities; Goodpaster, 2002; Machann et al., 2003; Karampatos et al., 2016). Due to its biochemical specificity, the use of magnetic resonance spectroscopy (MRS) is a valuable approach in measuring intramyocellular lipids. However, one of its limitations is high cost. In addition, while MRS can be done on many of the newer MRI machines, it requires more advanced work to set up and is unlikely to be available at most clinical facilities. It should also be noted that ultrasonography equipment is an emerging option for muscle tissue imaging. Ultrasound is commonly available at clinical centers, portable, and much cheaper than other imaging methods. However, like other common imaging methods, ultrasound has limitations in distinguishing between intramyocellular and extramyocellular fat.

While we are primarily relying on imaging techniques at this time, there is a pressing need for developing a rapid, easy to use, portable and cost-effective method for assessing myosteatosis in clinical settings. Fat infiltration in skeletal muscles increases with aging and influences muscle's metabolic and contractile function. A growing area of research on myosteatosis would benefit from a standardized approach to quantify myosteatosis across research studies. In addition, it is important to consider the population studied and the questions posed. More basic science and epidemiologic studies will be needed to determine the mechanisms responsible for increased myosteatosis with aging, as well as mechanisms underlying the effects of myosteatosis.

## Determinants of muscle quality: functional approaches to muscle quality estimates

While elements of muscle quality can be described using measures of muscle composition or myosteatosis, the impact of these muscle characteristics may be most relevant to mobility outcomes and the need for intervention in older adults. Muscle quality may be considered as a broad concept used to elucidate and describe muscle function, and it is largely influenced by the intricate intramuscular ultrastructure and morphology of contractile tissue. Structure-function relationships also affect muscle quality and can be described, in part, according to the “size principle” (Henneman, 1985). This principle holds that the functional potential of a skeletal muscle depends on the motor unit array and its sequential recruitment according to force demand. Functionally, the size principle of motor unit recruitment affects the tissue's capacity to perform its various functions such as generating force and aiding metabolism. Each of these functional roles may be impacted by changes in muscle composition and structure. Qualitative features of skeletal muscle tissue, such as composition, architecture, and morphology of the contractile apparatus translate into a variety of structural and functional indices of muscle quality (Fragala et al., 2015b).

### Functional consequences of myosteatosis

The functional impact of intermuscular fat has been demonstrated by several studies in which higher intermuscular fat was associated with poorer muscle function and strength (Goodpaster et al., 2001; Visser et al., 2002; Manini et al., 2007; Hilton et al., 2008; Delmonico et al., 2009). This appears to be related to a functional contractile hindrance within the muscle (Cesari et al., 2006; Lauretani et al., 2006; Schrager et al., 2007). A closer examination of muscle structure reveals how intramuscular fat may impede muscle function. With aging, the motor unit array may become compressed due to a decrease in both size and number of muscle fibers, particularly in type II fibers (Tomonaga, 1977; Larsson and Karlsson, 1978; Lexell et al., 1988). In fact, autopsy studies (Lexell et al., 1983) reveal 25% fewer muscle fibers in the medial vastus lateralis of older adults (72 ± 1 years) in comparison to younger individuals (30 ± 6 years). Moreover, aged skeletal muscle shows a variety of changes within the extracellular matrix, including collagen accumulation and altered elasticity (Kragstrup et al., 2011). The compressed motor unit array resulting from myosteatosis and non-contractile tissue accumulation may impede muscle activation and force production in older adults (Yoshida et al., 2012b).

In addition to muscle fiber size and number, biopsy studies also show changes in other qualitative characteristics of aged skeletal muscle such as changing fiber type distribution, in which the percentage and area of type II fibers in the vastus lateralis are reduced (Larsson and Karlsson, 1978). Accompanying changes in fiber type distribution within skeletal muscle are alterations in metabolism characterized by decreases in oxidative enzyme activity and muscle capillarization (Rogers and Evans, 1993). Examination of muscle biopsies of less functional or “clinically weak” muscle compared to “normal aged muscle” reveals qualitative differences in fiber packing, shape, intramuscular lipid distribution, and connective tissue (Fragala et al., 2014). Yet, despite the detailed characteristics of the individual muscle fibers (or cells) provided in muscle biopsy analysis, routine muscle biopsy procedures using the Bergstrom needle biopsy technique (Bergstrom, 1975) are invasive procedures not suitable for routine muscle quality assessment. Recent developments in fine needle biopsy techniques show promise for muscle histology assessment (Townsend et al., 2016) with far less pain and discomfort (Hayot et al., 2005).

### Muscle architecture

Cross-sectional muscle assessment yields important information about muscle function, as both individual muscle fiber diameter and cross sectional diameter of whole muscle are associated with muscle function as strength (Verdijk et al., 2010). Yet whole muscle contracts with the longitudinal shortening of muscle fibers by the sliding on the actin and myosin within the sarcomere units. Thus, assessment of both longitudinal and cross-sectional features of muscle architecture each provide important insights into muscle functioning. Ultrasonography enables the valid and reliable assessment of a variety of muscle architectural characteristics, such as anatomical cross-sectional area, muscle thickness, pennation angle, fascicle length, and echogenicity measured using grayscale analyses (i.e., a measure related to muscle density and myosteatosis; Cadore et al., 2012; Scott et al., 2012; Cartwright et al., 2013). Moreover, some evidence exists to show that local muscle hypertrophy may be detected on ultrasound before changes in DXA are apparent (Scanlon et al., 2014).

The muscle quality domain of force production is related to the architectural characteristics of skeletal muscle (Gans and de Vree, 1987; Gans and Gaunt, 1991; Fukunaga et al., 1997; Lieber and Friden, 2000), including muscle fiber length and arrangement in relation to the direction of force produced by the whole muscle (Gans and de Vree, 1987). Consequently, both cross sectional and longitudinal orientation measures of skeletal muscle have value in the evaluation of the size-strength relationship and the existence of age-related differences in muscle strength per size (Akagi et al., 2009). Longitudinal assessment by electron microscopic analysis of human skeletal muscle reveal sarcomeric changes in myofibrillar disorder, Z-line streaming, and dilatation in aged skeletal muscle (Scelsi et al., 1980). In consideration of such factors, architectural characteristics can be combined into a composite measure, often referred to as physiological cross-sectional area (PSCA)—a measure that is related to both leg extension strength and change in leg extension strength in older adults (Scanlon et al., 2014).

### Measures of muscle strength and function

#### Absolute and relative muscle strength

Measures of muscle composition, size, and architecture do not consider the neural input into the muscle fibers that dictate contraction potential and force production. Neural input is a critical component in muscle functioning, as cross-education models have shown increases in strength in an untrained limb through resistance training, without detectable changes in muscle size or the acute hormonal response (Beyer et al., 2016). According to the “Size Principle” of motor unit recruitment, functional measures of force output or strength reflect a muscle's ability to recruit fibers associated with motor unit arrays. Muscle weakness is collectively attributed to alterations in muscle composition, muscle contractile quality, and neural activation (Clark and Manini, 2010). As such, strength measures represent an important measure of muscle performance. This concept serves the basis of the FNIH model of sarcopenia, in which thresholds to define clinical weakness (i.e., grip strength <26 kg for men and <16 kg for women) have been associated with functional mobility consequences and incident mobility impairment (Alley et al., 2014; McLean et al., 2014; Studenski et al., 2014). In comparing grip to leg strength, both measures have been deemed suitable for screening for muscle weakness in older adults (Fragala et al., 2016). Moreover, strength measures show the ability to change in response to a variety of interventions for sarcopenia and frailty in older women with clinically meaningful muscle weakness, regardless of the presence of low lean mass (Fragala et al., 2015a). Recent work has further demonstrated the relation between the age-related decline in grip strength and forearm muscle quality (Abe et al., 2016). Muscle strength and size are commonly incorporated into a single measure of “muscle quality,” often referred to as relative strength. While little consensus exists on measures of strength and size that go into the equation, the common feature of the metric is the expression of muscle force production relative to muscle or body size. Like measures of muscle strength, measures of relative strength increase with resistance training intervention (Scanlon et al., 2014). Strength per unit of muscle tissue may provide a better indication of age-related differences in muscle quality prior to changes in lean tissue mass (Francis et al., 2016). Regardless, some evidence suggests that the relationship between relative strength and functional outcomes such as gait speed and chair rise may be restricted to older men, whereas BMI or adiposity appears to be the predictive factor in older women (Fragala et al., 2012; Straight et al., 2015).

#### Muscle quality index

**Figure 2:**
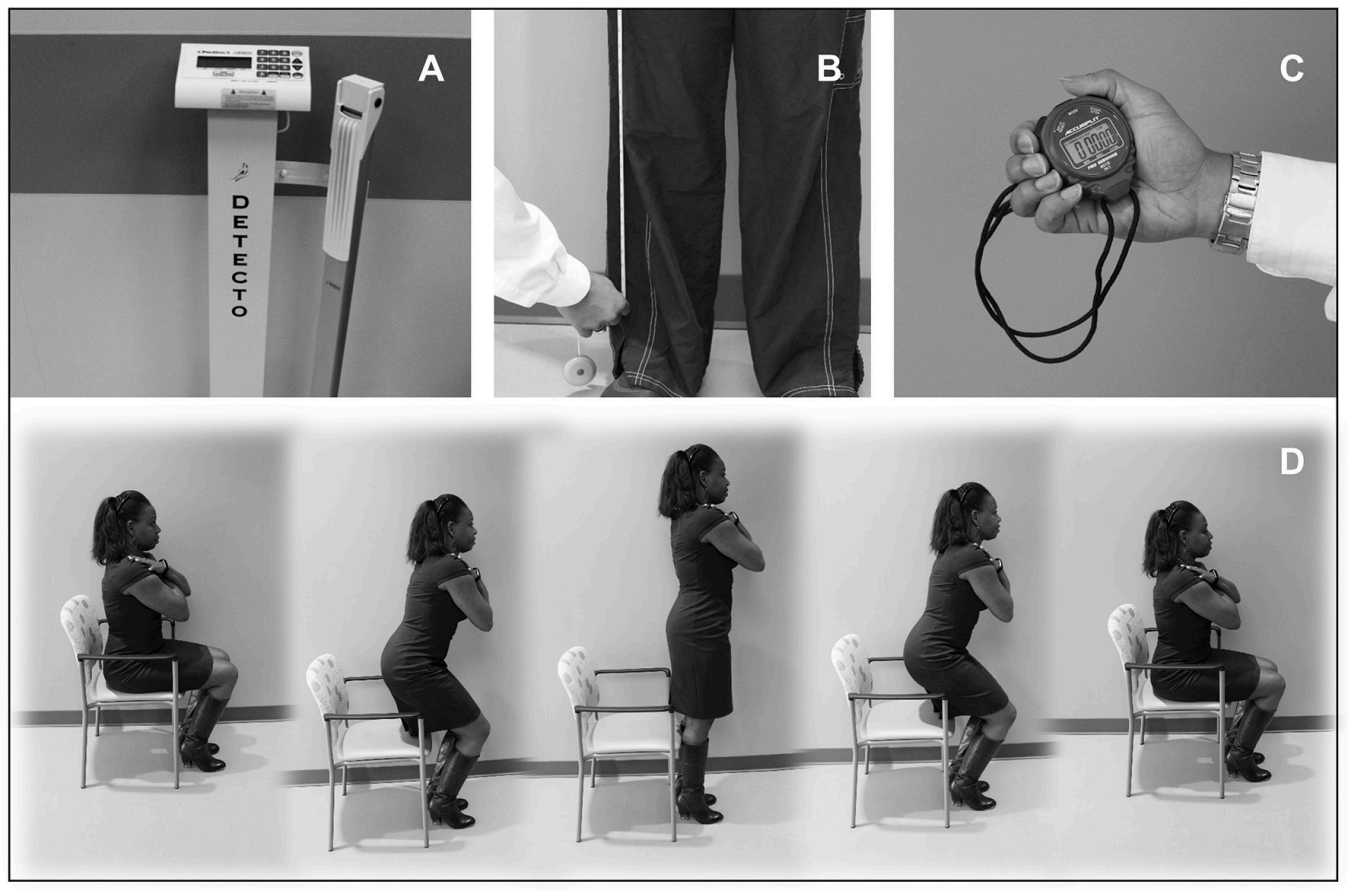
**A proposed performance-based index of muscle power. (A–D)** The Muscle Quality Index (MQI) is a performance-based functional assessment involving ten repetitions of the sit-to-stand maneuver performed as rapidly as possible. The test requires the use of a scale to record body mass **(A)**, a tape measure to obtain leg length **(B)**, along with a stopwatch and chair and for the timed functional task **(C,D)**. The MQI score is calculated using the following equation: ((leg length × 0.4) × body mass × gravity × 10)/sit-to-stand time.

Despite the value of relative strength measures, the required apparatus for some assessments such as PSCA continue to be difficult to incorporate into routine clinical assessment. As such, a variety of simple functional assessments have been explored for muscle function and elements of muscle quality such as grip strength, chair rise performance, and the timed get-up and go test, which have been shown to be predictive of subsequent mobility limitations (Guralnik et al., 1995). Importantly, consideration of body size enhances the interpretation of such tests. One novel functional metric that includes the features of a clinical test and simple body size measures has been referred to as the Muscle Quality Index (Takai et al., 2009), which provides a clinical measure of muscle power (Barbat-Artigas et al., 2012). This test is based on time to perform the simple chair rise. It also factors in body size and leg length into the equation. The Muscle Quality Index estimates muscle power from body anthropometrics and timed chair rises (Figure 2). It elaborates on the typical chair rise test by accounting for the anthropometric measures of body mass and leg length, which have been shown to alter the relationship between chair rise performance and leg strength (Takai et al., 2009). The validity and reliability of the Muscle Quality Index have been reported by Takai et al. (2009). In comparison to other functional measures (e.g., gait speed, grip strength, the get-up, and go test, etc.), the Muscle Quality Index has higher reliability and exhibits greater responsiveness following a resistance exercise regimen in older adults (Fragala et al., 2014).

The force production capacity of skeletal muscle may be the most prominent domain relating muscle quality to mobility outcomes. Skeletal muscle architecture characteristics may be significantly associated with force production capacity. In addition, simple functional tests such as the Muscle Quality Index may allow for muscle quality assessments in a variety of settings. Regardless of the myriad approaches, the need remains for consensus on the functional assessments of muscle quality that represent the contractile function of muscle tissue, are feasible in clinical environments, and are responsive to changes following intervention.

## Alternative clinical imaging measures of muscle quality

Tissue composition and morphology are elements of muscle quality that affect all domains of muscle function. Imaging modalities serve as a non-invasive approach to assess both tissue composition and body composition. Quantitative musculoskeletal diagnostic ultrasound has been proposed as a clinically viable means of characterizing muscle structure. Greater adoption of this sonographic method will occur if it helps the practitioner discriminate between older adults with and without sarcopenia and identify those at risk for impaired muscle performance.

### Muscle tissue composition as a window into muscle quality

Muscle mass is strongly coupled with muscle strength during the course of maturation through young adulthood. However, it has been observed that the relationship between muscle mass and strength decreases in magnitude as one ages, and that the age-related loss of muscle strength may occur more rapidly than the loss of LBM (Landers et al., 2001; Ferrucci et al., 2011). The measurement of muscle strength remains a vital outcome within the realm of geriatric assessment. Nevertheless, the isolated detection of low muscle strength cannot be used for staging or diagnosis, since there are varied causes of myogenic and neurogenic weakness that require additional medical follow up for the differential evaluation. Moreover, there is some evidence that diminished muscle tissue composition may precede losses in muscle mass as a contributing factor to the age-related decline in strength (Visser et al., 2005; Goodpaster et al., 2006; Watanabe et al., 2013; Ismail et al., 2015).

There are varied approaches to assessing the microscopic and gross morphological features of skeletal muscle tissue that may affect muscle quality. Muscle architecture consists of the macroscopic morphology of skeletal muscle based on the muscle shape and its fiber arrangement relative to the axis of force generation (Anneriet et al., 2007; Lieber, 2010). Whereas, muscle ultrastructure (also called, muscle microstructure) is the microscopic characterization of muscle tissue and its specialized cellular components and organization. This approach to myology dates back to Van Leeuwenhoek's light microscope studies in 1712 and was more fully elucidated in the seminal publication of the “Skeletal Muscle” Section of the *Handbook of Physiology* by the American Physiological Society in 1983 (Peachey et al., 1983). The study of muscle ultrastructure traditionally includes cytology, histology, surface morphology, and other anatomical approaches (Lieber, 2010; Eisenberg, 2011).

Regarding body composition assessment, multiple models exist which range from the molecular and cellular level to the tissue/organ and whole body level. However, all estimates of body composition are limited by a lack of true direct measurement (Heymsfield et al., 2015). Composition estimates are largely based on biochemical or tissue density qualities, with imaging methods typically incorporating a tissue/organ level of measurement (i.e., skeletal muscle tissue, adipose tissue, osseous tissue, visceral organs, and other tissues) or a whole-body level of measurement (i.e., head, trunk, and appendages) based on fat mass, fat-free mass, and bone mass. The use of imaging modalities as a non-invasive means of body and tissue composition assessment has a long history of usage in medical facilities. Although DXA, MRI, and CT are deemed acceptable tools for this purpose, significant barriers limit their incorporation into the sarcopenia staging process. DXA is perhaps the most widely accepted imaging modality for the estimate for LBM (expressed as total LBM or aLM/ht^2^) for sarcopenia staging, but it does not yield useful information concerning muscle quality based on adverse changes in muscle tissue composition. Both MRI and CT do provide estimates of muscle tissue composition, but CT is limited by its relative high ionizing radiation exposure, and MRI use is often beset by concerns related to access and cost. Standard segmental bioimpedance analysis (BIA) or refinements such as multi-frequency electrical impedance analysis could prove to be a non-imaging modality capable of characterizing elements of muscle quality. The phase angle derived from BIA has been associated with diminished strength in older adults and serial measures may partially reflect age-related changes in muscle tissue (Heymsfield et al., 2015). Both BIA and diagnostic ultrasound have comparative advantages, with the former having a low cost suitable for field use, and the latter having the additional ability to capture morphological features of skeletal muscle. Diagnostic ultrasound has been proposed as an alternative imaging modality for the use of sarcopenia screening and staging due to its portability, relatively low cost, and potential ability to provide proxy estimates of muscle tissue composition (Reimers et al., 1993; Sipilä and Suominen, 1993; Harris-Love et al., 2014).

### Quantitative sonography: considering the role of muscle tissue composition within the sarcopenia screening paradigm

Recent results from Cawthon and associates (Cawthon et al., 2015) involving the Osteoporotic Fractures in Men (MrOS) cohort were consistent with previously reported findings regarding the association of LBM with reported falls, limitations in mobility, and mortality. However, these investigators noted that staging individuals for sarcopenia using the major LBM cut off values only served to decrease the estimated risk of an adverse outcome in people within the cohort that had an event. The predictive value of LBM concerning morbidity and mortality in these community-dwelling older men did not exceed the predictive value of age alone. In contrast, findings from other studies involving large cohorts of community-dwelling older adults have indicated that muscle strength and muscle quality (based on ratios of strength and regional LBM, or tissue density algorithms involving Hounsfield units derived from CT scanning), but not muscle size or LBM, portend increased risk of hospitalization and incident disability (Cawthon et al., 2009; Hairi et al., 2010). Estimates of muscle quality based on CT scanning estimates of tissue density have been shown to be significantly associated with ultrasound echogenicity in samples of older women (Sipilä and Suominen, 1993). Moreover, preliminary evidence supports the observation that high echogenicity values obtained from anterior quadriceps ultrasound scans are associated with decrements in muscle force independent of muscle mass levels in older adults (Watanabe et al., 2013; Ismail et al., 2015).

**Figure 3:**
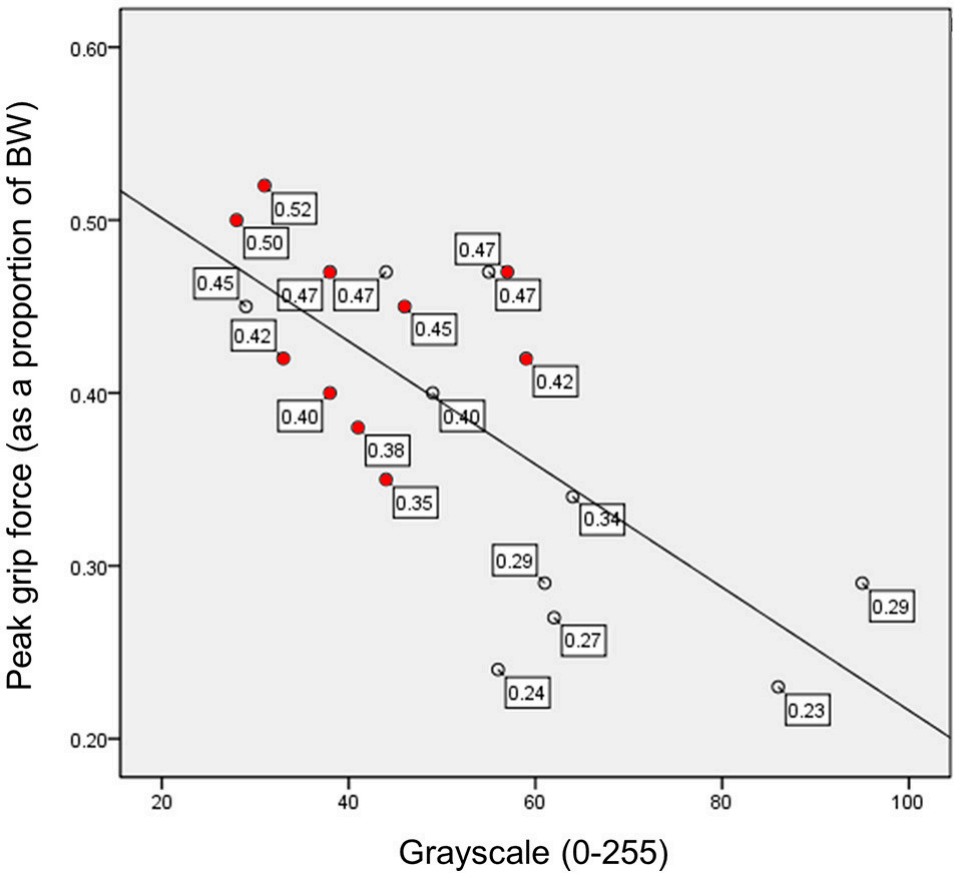
**Relative peak grip force and muscle echogenicity expressed in grayscale units**. Grayscale measures derived from the echogenicity of the rectus femoris have an inverse relationship with relative grip strength (peak force scaled to body weight). The filled data markers (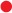) represent study participants with Class I sarcopenia (5.76–6.75 kg/m^2^) based on lean body mass estimates from DXA scanning, and the clear data markers (◦) represent study participants with normal body mass (>6.75 kg/m^2^). BW, body weight; scaled peak grip force, peak force in kg/body weight in kg; both scaled peak force and grayscale values are unitless measures.

A recent study featuring an assessment of muscle mass via DXA (e.g., aLM/ht^2^) and echogenicity as a proxy estimate of muscle tissue composition revealed that estimates of muscle quality via sonographic imaging were independently associated with relative grip strength, whereas LBM was not (Ismail et al., 2015). Upon accounting for both age and body fat in the relationship between echogenicity (as measured using grayscale histogram analysis; Harris-Love et al., 2016b) and strength, both echogenicity and age were identified as significant factors. However, echogenicity superseded age as a factor in the preliminary model (*r*_xyz_ = −0.52 vs. *r*_xyz_ = −0.38). Indeed, the study participants with higher levels of BMI and LBM (LBM > 6.75 kg/m^2^) exhibited lower levels of relative grip strength in comparison to those with lower levels of BMI and LBM with Class I sarcopenia (LBM = 5.76–6.75 kg/m^2^; Figure 3; Janssen et al., 2004a; Ismail et al., 2015). The collective observations of investigators (Visser et al., 2005; Goodpaster et al., 2006; Watanabe et al., 2013; Ismail et al., 2015) using imaging modalities to assess skeletal muscle in older adults raise the question, “Should an approach to muscle quality involving tissue composition be introduced as an independent criterion measure within the staging concept for pre-sarcopenia?” In order to address this question, additional studies should validate these reported observations in larger samples. These studies will allow better understanding of whether estimates of muscle tissue composition can be utilized as predictors of important clinical outcomes in older adults.

### Measurement considerations for image acquisition in quantitative sonography

Key factors that affect quantitative sonography procedures include the parameters associated with image acquisition, the methods used during image processing and analysis, the psychomotor skills of the examiner, and the wide array of technological capabilities among commercially available ultrasound machines. The selection of superficial muscle groups to determine sonographic estimates of LBM and muscle tissue composition confers some methodological advantages. Superficial muscle groups are readily identified via gross anatomical landmarks during the ultrasound examination. Also, their use minimizes excessive attenuation of the sound waves due to either large amounts of adipose tissue or the greater depth of penetration required for deeper tissues (Harris-Love et al., 2014). Typically, this approach to musculoskeletal imaging is aided by the use of high frequency linear ultrasound transducers which have a larger attenuation coefficient and may yield higher image resolution of the targeted muscle groups. The use of anterior axial and appendicular muscle groups have been proposed for ease of access during the ultrasound examination for sarcopenia staging (Ismail et al., 2015), but other approaches have been effectively used to derive estimates of LBM (Abe et al., 1994). Additional image acquisition parameters beyond the selected scanning sites may also foster imaging consistency. Many muscle groups with architectural features such a fusiform shape or convergent/triangular structure near the tendinous insertion may benefit from scanning in the longitudinal view. Previous findings from Zaidman and associates (Zaidman et al., 2008) indicated that longitudinal view images may be more reliable than the use of transverse view measurements when calculating calibrated muscle backscatter values from elbow flexor scans (intraclass correlation coefficients, ICCs, of 0.93–0.95 vs. 0.73–0.82). The method used to identify the region of interest (ROI) within the field of view may also impact estimates of muscle tissue composition using echogenicity values. While both near field and full muscle thickness ROI methods have been employed to characterize aberrant changes in echogenicity, the heterogeneity of age-related alterations in muscle tissue may merit the selection of the full muscle thickness ROI during image analysis (Watanabe et al., 2013; Harris-Love et al., 2016b).

Quantitative approaches to diagnostic musculoskeletal ultrasound have been shown to be reliable for the measurement of axial and appendicular muscle groups under controlled conditions (O'Sullivan et al., 2007; Sions et al., 2014). Moreover, investigators using quantitative sonography have exhibited good reliability for morphometry and for measures of echogenicity using grayscale histogram analysis and calibrated backscatter calculations (Zaidman et al., 2012, 2014). Nevertheless, the operator dependency of ultrasound measurements still exceeds the influence of the examiner in standard DXA and CT assessments of LBM. Although experience and training certainly increases scanning and image acquisition consistency between examiners, new modes of enhanced sonography have been proposed to promote reliability across examiners during serial imaging (Gilbertson and Anthony, 2013; Harris-Love et al., 2016a). Using a standard ultrasound transducer augmented with axial force sensors, examiners with varied levels of experience have demonstrated consistent material thickness measures over a range of applied scanning forces (Harris-Love et al., 2016a). Examiners with experience classifications of novice (1 month), intermediate (1 year), and experienced (>10 years) attained a high degree of interrater reliability (ICC_2, *k*_ = 0.97, *p* < 0.001; coefficient of variation = 1.5–2.9%) upon measuring a muscle mimetic ultrasound phantom utilizing up to 10 N of applied force. This demonstrated ability to obtain consistent image measurements at relatively high forces may have clinical implications given that the observed applied forces by sonographers may range from 5 to 14 N during abdominal examinations (Gilbertson and Anthony, 2013).

The development of enhanced force control scanning methods may continue to evolve and provide a viable alternative for sonographic imaging in varied clinical settings under realistic practice conditions. The technological advances of sonography are developing at a rapid pace in comparison to the contemporary innovations associated with DXA imaging. The continual improvement in sonography technology, coupled with the ongoing need to rectify image properties with tissue properties across different ultrasound devices, will be the source of opportunities and challenges in the quest for alternative imaging assessments of muscle quality.

## Measuring and controlling acquisition state in quantitative muscle sonography

Control systems have been engineered to address the major sources of variability in sonography and enhance its applicability to assess skeletal muscle quality. While this line of development has produced promising preliminary results, as discussed below, considerable efforts are still needed to better integrate technologies into systems that can help clinicians evaluate muscle quality. The measurement issues highlighted here, knowing and controlling the pose of a probe on the body and the amount of applied contact force—collectively referred to as the acquisition state—are central issues to reducing variability in the use of ultrasound to assess muscle quality.

### Sonographer and force variations

**Figure 4:**
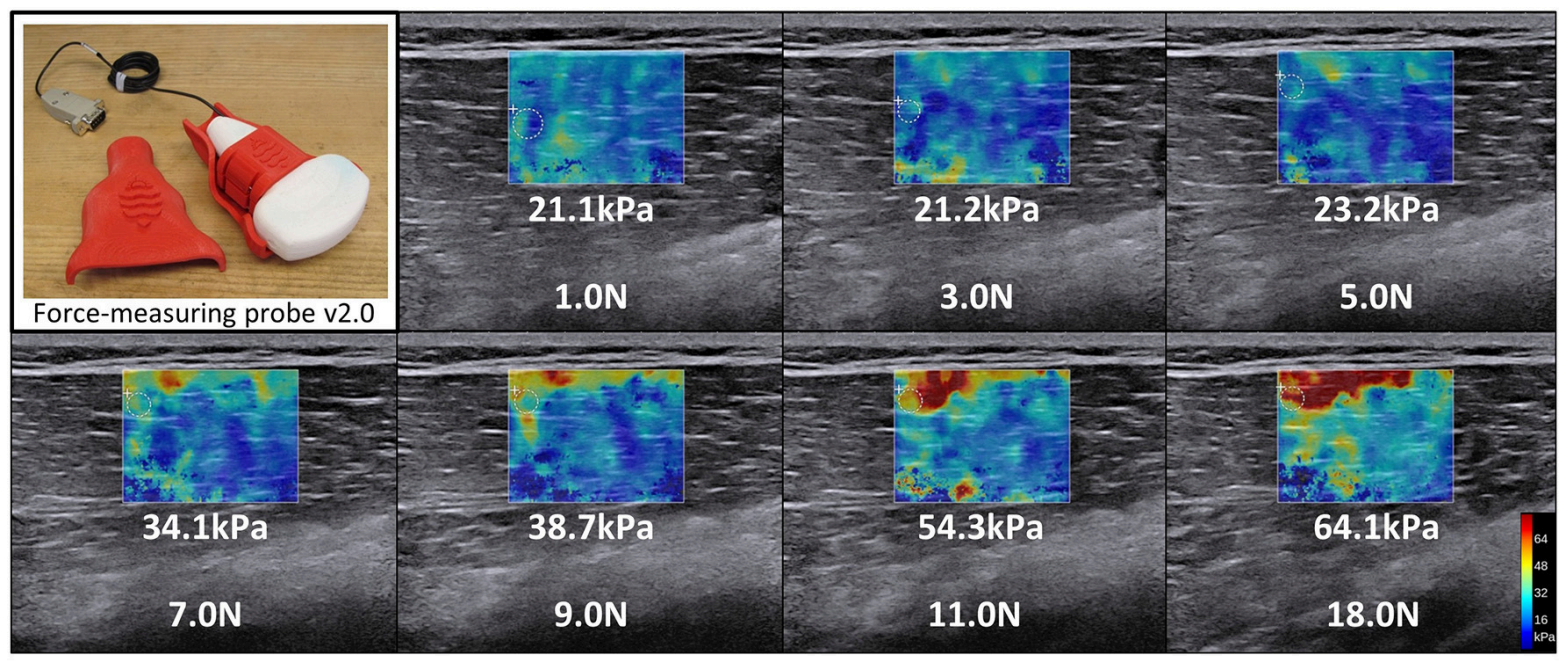
**Variation in shear wave elastography secondary to the applied scanning force**. Significant variation in shear wave elastography estimates of tissue Young's modulus shown in the figure is a function of preload differences typical of clinical sonography. The varying preload conditions depicted are typical of those seen across a range of operators in routine abdominal sonography and the resultant change in estimated tissue Young's modulus. This variation is explained by the observation that different bias compression levels pre-strain the tissue to different operating points along the tissue's non-linear stress-strain response. Estimated Young's modulus increases from 21.1 to 64.1 kPa in the vastus medialis as applied force (preload) increases from 1 to 18 N.

**Figure 5:**
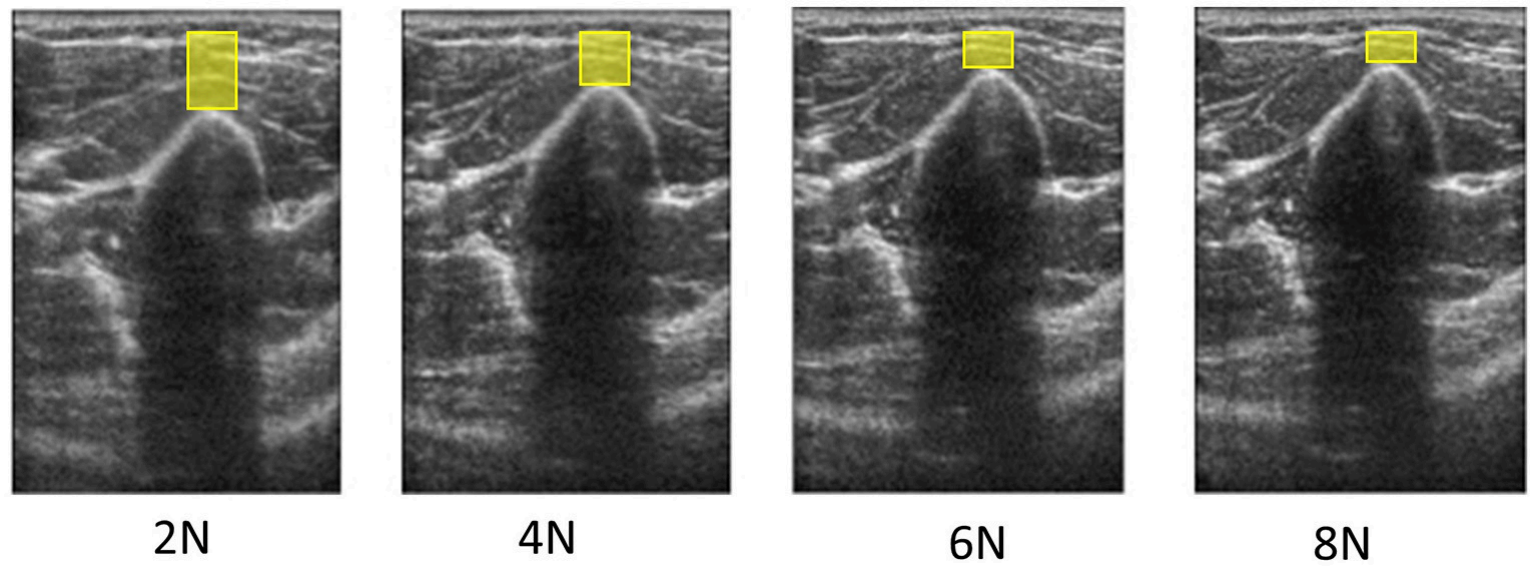
**Ultrasound images of the biceps from a healthy subject at four different forces**. Variation in the muscle thickness (denoted by the height of the yellow boxes), based on the measurement from the bone to the subcutaneous fat-muscle separation layer, is highly dependent on the examiner-generated force during scanning.

Variations in ultrasound probe contact force lead to variations in ultrasound images, making the images difficult to directly and quantitatively compare with each other. The contact force required to obtain an ultrasound image deforms the underlying soft tissue. Conventionally, the probe-patient contact force is controlled qualitatively by the human operator. A force-measurement platform (Figure 4) has been developed, which can simultaneously image and measure precise, operator-applied, force. During an ultrasound exam, it was demonstrated that contact forces exerted by the operator can vary, up to 50% over 30 s, resulting in images that are acquired at different levels of tissue deformation (Gilbertson and Anthony, 2013). This force measurement platform has been used to explore the impact of preload the on elastic property estimates of Young's modulus in tissue and to characterize the average preload applied during abdominal sonography (Dhyani et al., 2014); these values range between 4.4 and 10 N. This preliminary analysis reveals trends in applied force vs. years of experience and BMI of the patient. Shown in Figure 5 are ultrasound images of the biceps from a healthy subject, at four different applied forces. The muscle thickness as measured from the bone to the subcutaneous fat-muscle separation layer is highly dependent on force. Variable compression impacts the quantities extracted from ultrasound image analysis. Physical parameters such as elasticity are biased along the tissue's non-linear stress strain curve. Image-based parameters such as average grayscale level or and image texture are directly altered under different levels of tissue compression.

### Application of force-control to duchenne muscular dystrophy

Duchenne muscular dystrophy (DMD) is characterized by progressive disability leading to death and it remains one of the most common and devastating neuromuscular disorders of childhood (Bushby et al., 2010). It is caused by a genetic mutation that generates a complex sequence of events in muscle cells, which eventually undergo fibrosis and are replaced by adipose and connective tissue. Although a variety of promising new treatment strategies are in development, outcome measures for clinical trials remain limited for the most part to a set of functional measures, such as the 6-min walk test (Enright, 2003). While clearly useful, such measures are impacted by unrelated factors, such as mood and effort, and have limited repeatability. To address this and other limitations, magnetic resonance imaging is now being investigated as a surrogate measure (Willcocks et al., 2014). However, more easily applicable, cost-effective, office-based surrogate measures that provide high repeatability and sensitivity while still correlating strongly with disease status would find wider use. The information embedded in acquisition-state-controlled ultrasound images may provide convenient, non-invasive, clinically meaningful markers of muscle disease progression that surpass the functional measures currently in use.

Clinically meaningful markers of muscle disease progression in DMD are currently being developed. The potential for force-correlated ultrasound imaging to enable automated classification of muscle tissues structures in individuals with DMD has been shown. Image-based quantities, such as variance maps (Koppaka et al., 2014a), and image texture (Koppaka et al., 2014b), have been used in classification schemes. Automated classification was found to be improved when the biomarkers extracted (from the quadriceps, biceps, forearm, tibialis anterior, and medial gastrocnemius) were tagged with force information. Similar methods may be used to promote ultrasound as a quantitative non-invasive tool for not only classifying disease severity, but also for tracking progression over time.

### Force variation in elastography

Ultrasound elastography, whether strain elastography or shear wave elastography (SWE), also suffers from operator-dependent and acquisition state variation discussed above. Ultrasound elastography limitations have been identified by scientists and clinicians in the context of technique performance, clinical utility, and work-flow. The most frequently acknowledged limitation is repeatability and reproducibility. A few confounding factors introduce bias and variation in stiffness measurement, including operator dependence, concerning both strain elastography and SWE, and cross-vendor system dependence of SWE caused by different shear wave excitation mechanisms, tissue mechanical models, and signal processing involved in stiffness reconstruction. Before ultrasound elastography can be fully adopted as a routinely used clinical tool for diagnosis and therapy monitoring, these technical and clinical challenges must be overcome by the entire industry.

Soft tissue is mechanically non-linear, meaning that elastography measurement varies with the operator-applied transducer force (i.e., preload). Preload-induced variation may lead to inaccurate diagnosis and poses challenges to establishing measurement standardization and clinical guidelines. Even with SWE, which is considered more operator-independent than strain elastography, stiffness measurement on the same tissue can vary significantly. The results in Figure 4 show significant variation in shear wave elastography estimates of tissue elasticity as a function of preload differences typical of clinical sonography. The varying preload conditions depicted, 1–18 N, are typical of those seen across a range of operators in routine sonography (Dhyani et al., 2014) and the resultant change in estimated tissue Young's modulus ranged from 21.1 to 64.1 kPa in the vastus medialis. This variation is explained by the observation that different bias compression levels pre-strain the tissue to different operating points along the tissue's non-linear stress-strain response.

### Position and orientation variation: skin as a body location encoding system

Knowing and controlling the position and orientation of an ultrasound probe on the body is also a central issue to reducing variability over serial examinations. Local skin appearance can be used to help relocate a probe on the body. This is accomplished by attaching a small camera to an ultrasound probe and capturing high magnification images of the skin at the same time as the ultrasound images.

**Figure 6:**
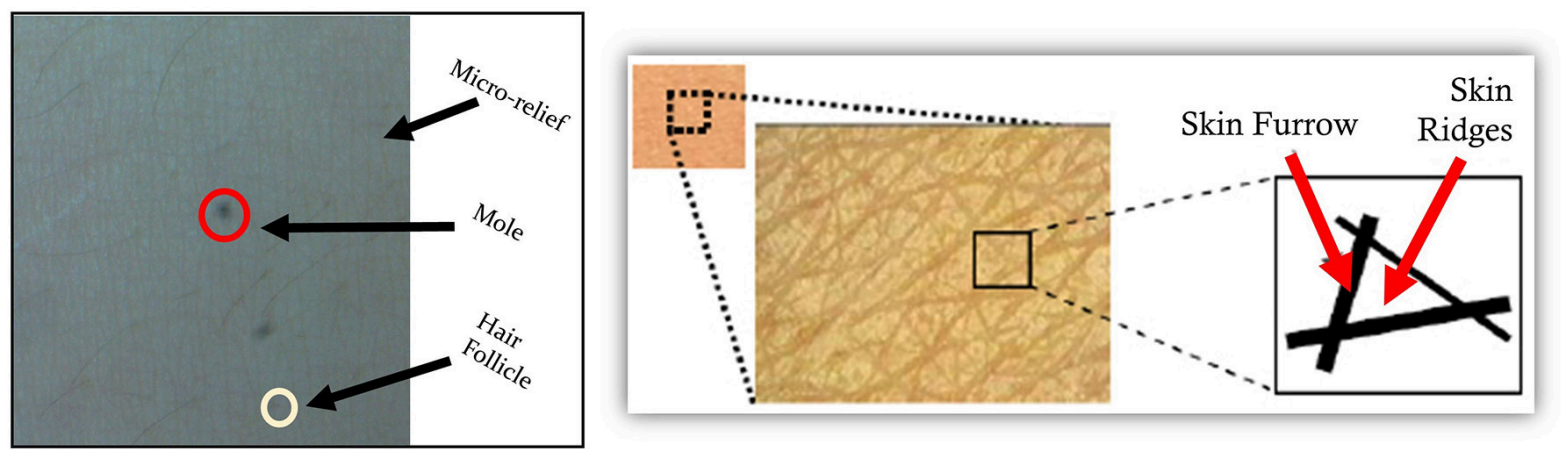
**Sound transducer localization using unique skin features**. A freehand ultrasound platform featuring an optical camera kinematically coupled to an ultrasound transducer may be used with software that extracts unique skin features and provides transducer localization relative to the skin features. The ultrasound transducer registration facilitates the acquisition of three-dimensional ultrasound volume estimates derived from a standard optically tracked two-dimensional sonograms. This approach allows for scanned images to be generated and reformatted in any plane to allow for the comparison of matched images across serial examinations.

Patterns of skin features are unique when viewing a sufficiently large area of skin and include micro reliefs, follicles, moles, and melanin variation (Figure 6). This uniqueness allows skin to be used as a relative-motion or absolute-location encoding system for the body. Based on this concept, a freehand ultrasound platform has been developed which localizes an ultrasound probe in 6-degree-of-freedom (DOF) with respect to the patient (and not room coordinates) by tracking natural skin features (i.e., fiducial-free) with a small camera mounted to the ultrasound probe (Sun and Anthony, 2012; Sun et al., 2013a,b). The system comprises: (1) an optical camera kinematically coupled to an ultrasound transducer, (2) software that extracts unique skin features, and (3) software that localizes the transducer by tracking the skin features (Sun and Anthony, 2012; Sun et al., 2013a,b, 2014).

The process involves simultaneously recording, synchronously in time, ultrasound probe images inside the body and camera images of the skin. Skin features are used to track relative motion along the body. The same skin features can be used to virtually identify and tag unique locations on the body; this system permits placement of the ultrasound transducer in the same body location and orientation by different operators on serial examinations. In practice, multiple sources of information, e.g., camera images and ultrasound images and known regularities motion smoothness, are combined for optimal probe pose estimation. In this way, a body-contoured skin map is constructed and each ultrasound image is accurately localized with respect to the map—a process termed simultaneous localization and mapping (SLAM; Thrun and Leonard, 2008).

This process results in a spatially accurate three-dimensional (3D) ultrasound volume derived from a standard optically tracked two-dimensional sonogram. Consequently, an ultrasound probe may be approximately registered/relocated at a known location and orientation with respect to patient coordinates. The 3D volume could be used to generate multi-planar images which should be nearly indistinguishable from directly acquired ultrasound images (Sun et al., 2014). Sonograms can be reformatted in any plane, allowing comparison of matched images across serial examinations. The re-registration ability should improve the ability to longitudinally monitor near-surface or fixed location organs or tissue structures.

Along with improvement of the hardware technologies and better integration into systems, significant research is needed to further develop, refine, and validate analysis algorithms and workflow software. These improvements would support the effort to produce automated or augmented quantitative analyses of ultrasound imagery applied to skeletal muscles. The rapid technological advancements in ultrasound systems will continue to have a meaningful impact on the assessment of muscle quality, and perhaps expand the methods used for the screening and staging of sarcopenia and other neuromuscular disorders.

## Symposium summary

The developing framework for assessing muscle quality allows investigators to move away from a strict LBM-driven assessment approach to sarcopenia research, and may help to end the confusion between “measurement methods” and specific “domains” of muscle quality. However, the need continues to reach consensus on standardized approaches to assessing aspects of muscle quality that influence the major functions of skeletal muscle and are likely part of SMFD. Some important messages to consider as research efforts evolve include:

- The existent body of literature concerning myosteatosis supports the premise that excessive ectopic fat depots in muscle and intra-myocellular lipids impact both functional decline and metabolic dysfunction in older adults.
- While current consensus-driven sarcopenia staging algorithms relates to the loss of muscle mass, low strength, and diminished functioning, the larger concept of age-related muscle dysfunction may need to incorporate metabolic health independent of mobility status or muscular strength.
- A measured approach to scaling factors regarding timed tests of performance and force production may add value to the clinical assessment of sarcopenia and the monitoring of patient status following exercise-based interventions and other forms of treatment.
- Quantitative diagnostic sonography and multi-frequency electrical impedance analysis are clinically feasible approaches to estimating tissue composition. These assessment tools may aid the assessment of muscle quality in a variety of settings. However, additional validation studies remain to be done in order to move toward a wider adoption of these alternate methods.

The current sarcopenia screening, staging, and classification criteria should be open to periodic revision in light of the continued evolution of performance-based testing and the advances in imaging technology to assess muscle quality. Also, features of skeletal muscle related to tissue characteristics may ultimately serve to aid the sarcopenia diagnosis if such outcomes exhibit utility in discriminating between patient groups and predicting the incidence of metabolic disorders or disablement. However, it is important to note that this symposium report has limitations regarding the scope of the topics presented. Other causative factors, such as reactive oxygen species (ROS), negatively impact muscle quality and merit further consideration. ROS plays an extremely important role in all muscle functions and is closely related to muscle aging, contraction, fatigue, dystrophy or waste, but this topic was beyond the goals of symposium (Zuo et al., 2012, 2013, 2015; Min et al., 2015). Finally, garnering consensus on the major domains of muscle quality, and measurement methods suitable for a variety of settings, remains a priority. Addressing the aforementioned concerns may serve to advance skeletal muscle research in the context of skeletal muscle function deficit and help identify individuals who would benefit from interventions to improve muscle quality.

## Author contributions

RC, MH, IM, MF, BA, and TM drafted, edited, and revised the manuscript; all authors approved final draft of the manuscript prior to publication.

## Funding

This publication was partially supported by the VA Office of Research and Development—Rehabilitation R&D Service (1IK2RX001854-01). The funders had no role in study design, data collection and analysis, decision to publish, or preparation of the manuscript.

### Conflict of interest statement

The authors declare that the research was conducted in the absence of any commercial or financial relationships that could be construed as a potential conflict of interest. The reviewer ZL and handling Editor declared their shared affiliation, and the handling Editor states that the process nevertheless met the standards of a fair and objective review.
